# Incubation period of *Clostridioides difficile* infection in hospitalized patients and long-term care facility residents: a prospective cohort study

**DOI:** 10.1017/ash.2024.392

**Published:** 2024-09-24

**Authors:** Scott R. Curry, Michelle T. Hecker, Justin O’Hagan, Preeta K. Kutty, Yilen K. Ng-Wong, Jennifer L. Cadnum, Heba Alhmidi, Melany I. Gonzalez-Orta, Carlos Saldana, Brigid M. Wilson, Curtis J. Donskey

**Affiliations:** 1 Division of Infectious Diseases, Medical University of South Carolina, Charleston, SC, USA; 2 Division of Infectious Diseases, MetroHealth Medical Center, Cleveland, OH, USA; 3 Department of Medicine, Case Western Reserve University School of Medicine, Cleveland, OH, USA; 4 Division of Healthcare Quality Promotion, National Center for Emerging and Zoonotic Infectious Diseases, Centers for Disease Control and Prevention, Atlanta, GA, USA; 5 Research Service, Louis Stokes Cleveland VA Medical Center, Cleveland, OH, USA; 6 Geriatric Research, Education, and Clinical Center, Louis Stokes Cleveland VA Medical Center, Cleveland, OH, USA

## Abstract

**Background:**

The incubation period for *Clostridioides difficile* infection (CDI) is generally considered to be less than 1 week, but some recent studies suggest that prolonged carriage prior to disease onset may be common.

**Objective:**

To estimate the incubation period for patients developing CDI after initial negative cultures.

**Methods:**

In 3 tertiary care medical centers, we conducted a cohort study to identify hospitalized patients and long-term care facility residents with negative initial cultures for *C. difficile* followed by a diagnosis of CDI with or without prior detection of carriage. Cases were classified as healthcare facility-onset, community-onset, healthcare facility-associated, or community-associated and were further classified as probable, possible, or unlikely CDI. A parametric accelerated failure time model was used to estimate the distribution of the incubation period.

**Results:**

Of 4,179 patients with negative enrollment cultures and no prior CDI diagnosis within 56 days, 107 (2.6%) were diagnosed as having CDI, including 19 (17.8%) with and 88 (82.2%) without prior detection of carriage. When the data were censored to only include participants with negative cultures collected within 14 days, the estimated median incubation period was 6 days with 25% and 75% of estimated incubation periods occurring within 3 and 12 days, respectively. The observed estimated incubation period did not differ significantly for patients classified as probable, possible, or unlikely CDI.

**Conclusion:**

Our findings are consistent with the previous studies that suggested the incubation period for CDI is typically less than 1 week and is less than 2 weeks in most cases.

Understanding the incubation period, defined as the time between colonization and the onset of symptoms, is important for the surveillance and control of infectious diseases.^1^ For *Clostridioides difficile*, studies conducted in the 1990s provided evidence that the incubation period is usually short with onset of diarrhea within 1 week after acquisition of colonization.^2,3^ Samore et al^2^ found that the time from the first positive culture to the onset of diarrhea was <10 days for 8 of 9 hospitalized patients developing *C. difficile* infection (CDI) (median incubation period, 3 days). In contrast, Curry et al^4^ recently reported that the time from new detection of colonization to the onset of symptoms was 8–28 days for 7 CDI patients with genetically related colonizing and infecting strains. Other recent studies have demonstrated that many patients diagnosed as having healthcare-associated CDI are asymptomatically colonized with *C. difficile* on admission, suggesting the possibility of prolonged carriage prior to disease onset or false-positive diagnosis of CDI in patients with diarrhea due to other causes (eg, laxatives).^5,6^

Given the conflicting evidence regarding the incubation period for *C. difficile*, there is a need for additional studies in high-risk patient populations. Therefore, we conducted a multicenter cohort study including hospitalized patients and long-term care facility (LTCF) residents. Our primary goal was to estimate the incubation period for CDI in patients with initial negative cultures who subsequently developed an infection.

## Methods

### Setting

The study was conducted in 3 tertiary care facilities, including the Cleveland VA Medical Center, MetroHealth Medical Center (Cleveland, Ohio), and the Medical University of South Carolina. At the Cleveland VA Medical Center and MetroHealth Medical Center, enrollment sites included the hospital and affiliated LTCFs providing postacute and residential care with 250 and 200 beds, respectively. One objective of the study was to examine the natural history of *C. difficile* colonization and infection after new acquisition of carriage; those findings were published elsewhere.^7^ At the time of the study, clinical testing for CDI at MetroHealth Medical Center was performed using a 2-step C*. difficile* testing algorithm with an initial nucleic acid amplification test (NAAT) followed by testing of NAAT-positive specimens with an enzyme immunoassay (EIA) for the toxin. The other facilities used a standalone NAAT test for CDI testing.

### Study design

Between November 1, 2016, and November 3, 2018, we conducted a cohort study in the 3 facilities to estimate the incubation period for CDI. The timing of subject enrollment varied for each facility. The Cleveland VA Medical Center for a 1-year period, whereas MetroHealth Medical Center and the Medical University of South Carolina enrolled participants for 6-month periods. The institutional review board of each hospital approved the study protocol.

At each facility, participants were screened for asymptomatic carriage by culturing perirectal swab specimens for toxigenic *C. difficile* (ie, culture with confirmation of toxin production using EIA for the toxin).^5,7^ At the Cleveland VA Medical Center and MetroHealth Medical Center, hospital or LTCF residents with an anticipated length of stay of 3 or more days at the time of admission were enrolled. Research personnel collected perirectal swabs on admission and then weekly while in the hospital or LTCF or during scheduled outpatient clinic visits when feasible. For participants with new detection of colonization by toxigenic *C. difficile*, additional swabs were collected monthly for up to 6 months. At the Medical University of South Carolina, infection control personnel collect perirectal swabs on admission and weekly from inpatients in 10 hospital wards for vancomycin-resistant *Enterococcus* (VRE) screening; for this study, the perirectal swabs collected for admission and weekly screening between April 3, 2018, and November 3, 2018, were cultured for toxigenic *C. difficile*.

Participants were followed for diagnosis of CDI during the enrollment period and for 3 months after completion of enrollment. For all study participants diagnosed as having CDI, the stool specimen used for diagnosis was cultured for toxigenic *C. difficile*, and ribotyping was performed for a subset of isolates. A medical record review was conducted to obtain information on demographics, medical conditions, medications, prior CDI, and site of enrollment. Electronic data capture tools (REDCap 13.4.13) were used to collect this information at the Medical University of South Carolina.^8^ NAAT-positive cases were classified as probable, possible, or unlikely CDI by the lead investigator for each hospital using a modification of the classification categories of Hecker et al^9^ Probable CDI cases had ≥3 unformed stools per day with no alternative explanation and had received antibiotics or chemotherapy. Unlike Hecker et al^9^, we did not classify patients with ≥5 unformed stools per day and leukocytosis or radiographic findings consistent with CDI as probable cases if they did not have prior antibiotic or chemotherapy exposure. Possible CDI cases had ≥3 unformed stools per day with no alternative explanation but with no prior antibiotic or chemotherapy exposure. Unlikely CDI cases had >3 unformed stools per day but with a definite alternative explanation (eg, laxatives) or <3 unformed stools per day and no leukocytosis or radiographic findings consistent with CDI or ileus.

### Microbiology and molecular typing

Perirectal swabs and stool specimens were cultured for toxigenic *C. difficile* as described previously.^7^ For perirectal swabs, polymerase chain reaction (PCR) analysis has previously been reported to have 68% sensitivity in comparison to toxigenic culture.^10^ For stool specimens used for diagnosis of CDI, PCR analysis has been reported to have >90% sensitivity in comparison to toxigenic culture.^11^ PCR ribotyping was completed for a subset of the isolates recovered from patients developing CDI.^12^

### Epidemiologic definitions and statistical analysis

CDI cases were classified as healthcare facility-onset (HO) CDI, community-onset, healthcare facility-associated (CO-HCFA) CDI, or community-associated CDI.^13^ For the estimation of the incubation period, only participants with a negative initial culture and no prior CDI diagnosis within 2 months (56 days) were included. The exclusion of patients with CDI within 56 days was based on the surveillance definition classifying CDI cases occurring within 56 days after the onset of a previous CDI case as a recurrence provided that CDI symptoms from the earlier case resolved.^13^ For the purposes of the study, the incubation period was defined as the time between detection of colonization and diagnosis of CDI; the date of diagnosis was used rather than the date of onset of symptoms because the timing of symptom onset was not available for the patients from the Medical University of South Carolina. For participants developing CDI without prior detection of colonization, the incubation period was defined as the time from the day of the most recent negative culture to the day of CDI diagnosis.

A parametric accelerated failure time model was used to estimate the distribution of the incubation period. Analysis of the incubation period was performed using the methods of Reich et al^14^ with the coarseDataTools package in R 3.5.0. (R Foundation for Statistical Computing, Vienna, Austria).^15^ All data were doubly interval censored. Analyses were performed on 3 populations: one including all CDI cases and 2 subgroups of cases including only patients with an interval of ≤30 or ≤14 days from a negative swab to CDI diagnosis. The rationale for the 2 subgroup analyses was that many patients at the Medical College of South Carolina site had cultures collected intermittently during separate hospital admissions. It was reasoned that the inclusion of negative baseline cultures long before the CDI diagnosis in the absence of subsequent follow-up cultures might result in an overestimation of the incubation period. Model fit for all 3 subgroups was compared under assumed log-normal, Gamma, and Weibull distributions.

## Results

**Figure 1.**
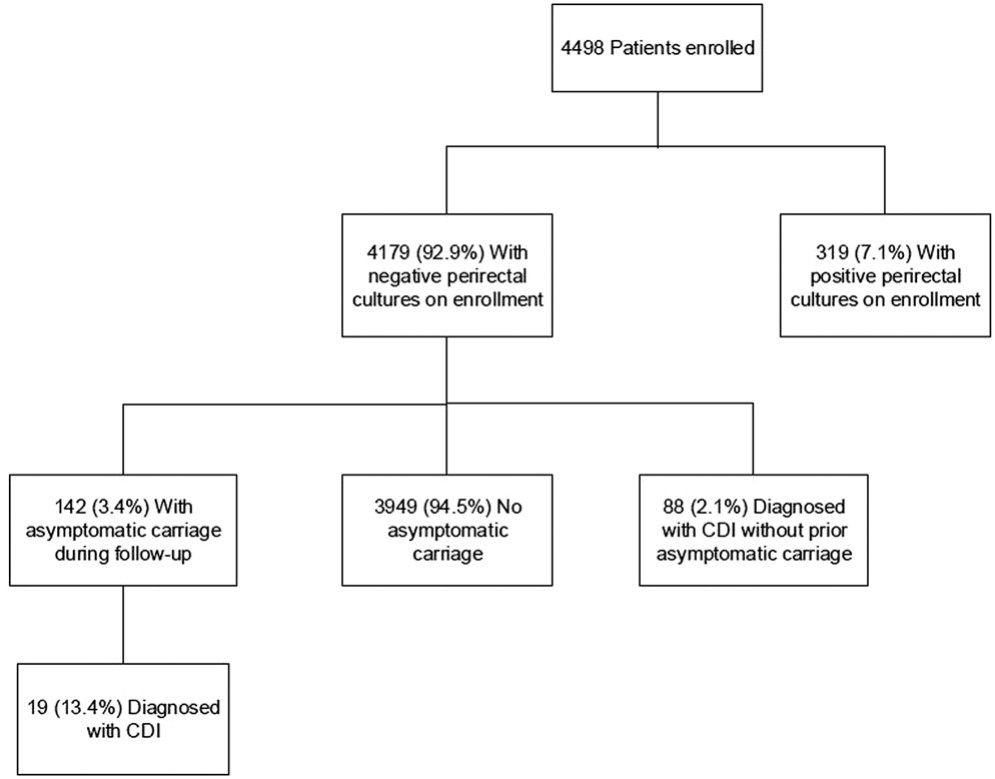
Flow diagram for the study participants. CDI, *Clostridioides difficile* infection.

Figure 1 provides a flow diagram for the study participants. Of 4498 total patients enrolled from the 3 facilities (400 at the Cleveland VA Medical Center, 116 at MetroHealth Medical Center, and 3982 at the Medical University of South Carolina), 4179 (92.9%) had negative cultures on enrollment. The percentage of patients with 1 or more follow-up perirectal cultures was substantially lower at the Medical University of South Carolina versus the Cleveland VA Medical Center and MetroHealth Medical Center (28% versus 84% and 88%, respectively), primarily due to discharge prior to follow-up culture collection. One-hundred-seven (2.6%) of those with negative enrollment cultures and no prior CDI diagnosis within 56 days were subsequently diagnosed as having CDI during the study period, including 12 at the Cleveland VA Medical Center, 2 at MetroHealth Medical Center, and 93 at the Medical University of South Carolina. Seven of 14 (50%) diagnosed as having CDI at the Cleveland VA Medical Center and MetroHealth Medical Center were nursing home residents. Information on nursing home residence prior to hospital admission was not available for the Medical University of South Carolina.

Table 1 shows the characteristics of the participants diagnosed as having CDI. Eighty-seven (81.3%) of the patients were classified as having probable or possible CDI, and 88 (82.2%) were HO or CO-HCFA cases. Of the 107 patients diagnosed as having CDI, 100 (93.5%) had received antibiotics within 90 days of the CDI diagnosis, and 100 (93.5%) were hospitalized at the time of enrollment. For the 24 CDI isolates subjected to ribotyping, the ribotypes were F014-020 (*N* = 5), F027 (*N* = 4), F106 (*N* = 3), FP407 (*N* = 3), F103 (*N* = 3), F015 (*N* = 2), F087 (*N* = 1), FP494 (*N* = 1), and F001 (*N* = 1) and a pattern with no match in the reference database (*N* = 1).^10^

**Table 1.** Baseline characteristics of the 107 patients diagnosed as having *Clostridioides difficile* infection after an initial negative culture

Characteristic	Value
**Baseline**	
Age in years, mean (range)	59.5 (18.2, 91.0)
Male sex	53 (49.5)
Previous hospitalization within 90 days	68 (63.6)
Previous CDI more than 56 days before enrollment^a^	5 (4.7)
Antibiotic treatment within 90 days	100 (93.5)
Site of enrollment	
Hospital	100 (93.5)
Long-term care facility	7 (6.5)
Medical conditions	
Chronic lung disease	23 (21.5)
Diabetes mellitus	37 (34.6)
Cancer	48 (44.9)
Cerebrovascular accident	8 (7.5)
Major surgery within 90 days	42 (39.3)
Cirrhosis	8 (7.5)
End-stage renal disease	8 (7.5)
Spinal cord injury	1 (0.9)
Solid organ or bone marrow transplant	23 (21.5)
MRSA colonization	23 (21.5)
VRE colonization	20 (18.7)
Maximum WBC −3 to +7 days from CDI diagnosis (mean, range)^b^	12.6 (0, 42.8)
Lowest serum albumin −3 to +7 days from CDI diagnosis (Mean, range)^c^	2.4 (1.1, 4.8)
CDI surveillance classification^d^	
Healthcare facility-onset	62 (57.9)
Community-onset, healthcare facility-associated	26 (24.3)
Community-associated	19 (17.8)
Clinical likelihood of CDI^e^	
Probable	57 (53.3)
Possible	30 (28.0)
Unlikely	20 (18.7)

Note. CDI, *Clostridioides difficile* infection; MRSA, methicillin-resistant *Staphylococcus aureus*; VRE, vancomycin-resistant *Enterococcus*; WBC, white blood cell. Data are no. (%) of patients, unless otherwise specified.

a Patients with prior CDI cases within 56 days were excluded from the analysis;

b excludes 10 patients with missing data;

c excludes 23 patients with missing data;

d CDI surveillance definitions as defined in Kociolek LK, et al^11^;

e probable cases had ≥3 unformed stools per day with no alternative explanation and had received antibiotics or chemotherapy; possible cases had >3 unformed stools per day with no alternative explanation but with no prior antibiotic or chemotherapy exposure; unlikely cases had >3 unformed stools per day but with a definite alternative explanation (eg, laxatives) or <3 unformed stools per day and no leukocytosis or radiographic findings consistent with CDI or ileus.

Of 107 patients developing CDI after an initial negative culture, 19 (17.8%) had prior detection of colonization, and 88 (82.2%) developed CDI with no prior detection of carriage (Figure 1). For the 88 patients developing CDI with no prior detection of carriage, the mean number of prior negative perirectal cultures was 2 (range, 1–10), and 63 (71.6%) and 53 (60.2%) had negative cultures collected within 30 and 14 days of the CDI diagnosis, respectively. For the 19 patients developing CDI with prior detection of carriage, the mean number of prior positive perirectal cultures was 1.3 (range, 1–3), and the median time from the initial positive culture to the diagnosis of CDI was 10 days (range, 1–53 days). Of 4 CDI patients with ribotyping completed on paired isolates, 4 (100%) had matching ribotypes for prior colonization and infection isolates.

**Figure 2.**
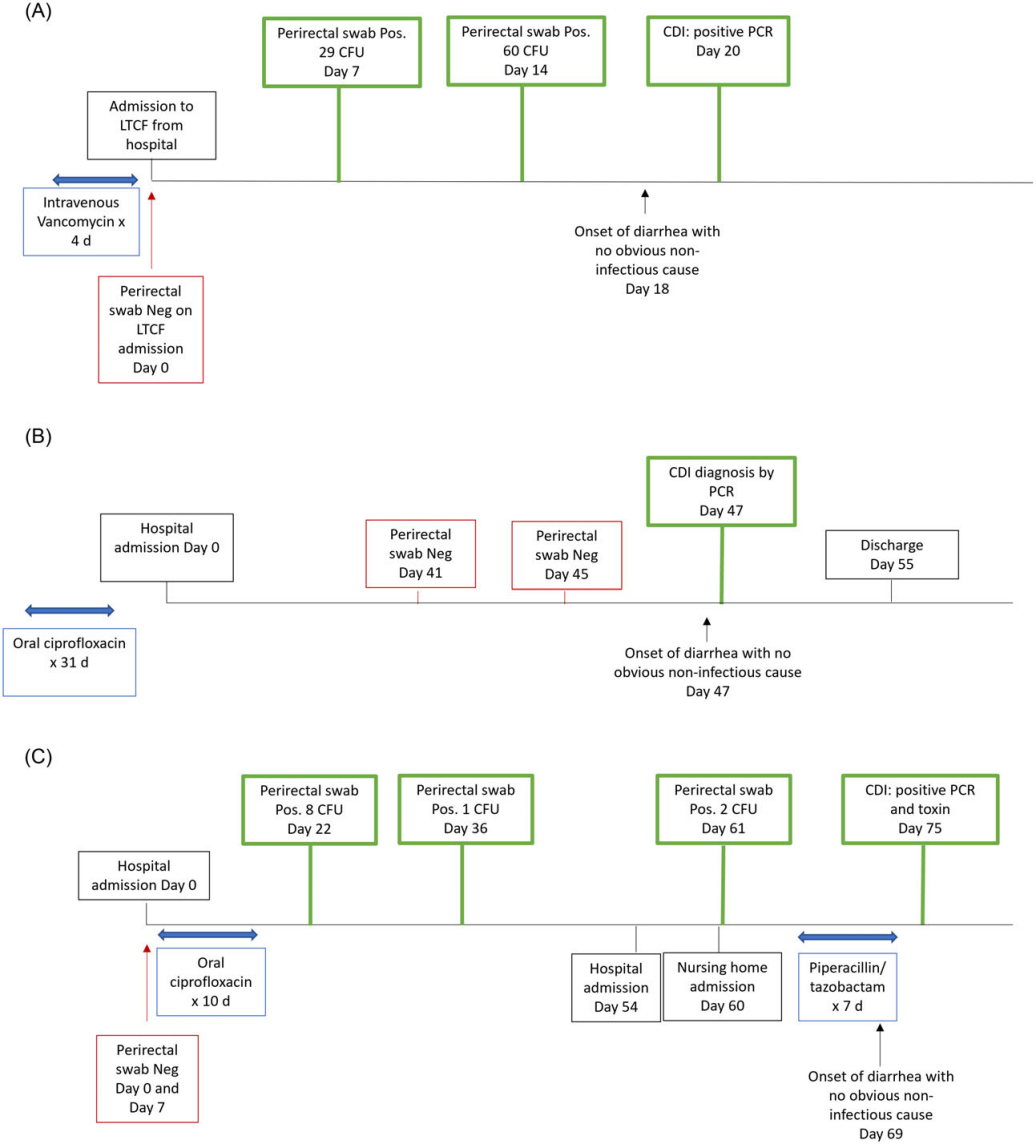
Examples of patients diagnosed as having *Clostridioides difficile* infection (CDI) with (A, C) and without (B) prior detection of asymptomatic carriage. A, Diagnosis of CDI with a ribotype F027 strain with detection of the carriage 13 days prior; B, Diagnosis of CDI with a ribotype F014-020 strain with no prior detection of carriage (estimated incubation period 2 days); C, Diagnosis of CDI with a ribotype F014-020 strain with detection of the carriage 53 days prior. LTCF, long-term care facility; CFU, colony-forming units; PCR, polymerase chain reaction; Neg, negative.

Figure 2 shows examples of patients diagnosed as having CDI with and without prior detection of carriage with estimated incubation periods of 13, 2, and 75 days. The patient in 2.A acquired colonization with a ribotype F027 strain on day 7 of hospitalization and was diagnosed as having CDI on day 20; no noninfectious cause of diarrhea was identified. The patient in 2.B was diagnosed as having CDI due to a ribotype F014-020 strain with no prior detection of colonization. The patient in 2.C acquired and maintained persistent colonization with a ribotype F014-020 strain and was diagnosed as having CDI based on positive PCR and enzyme immunoassay for toxin 53 days later.

**Figure 3.**
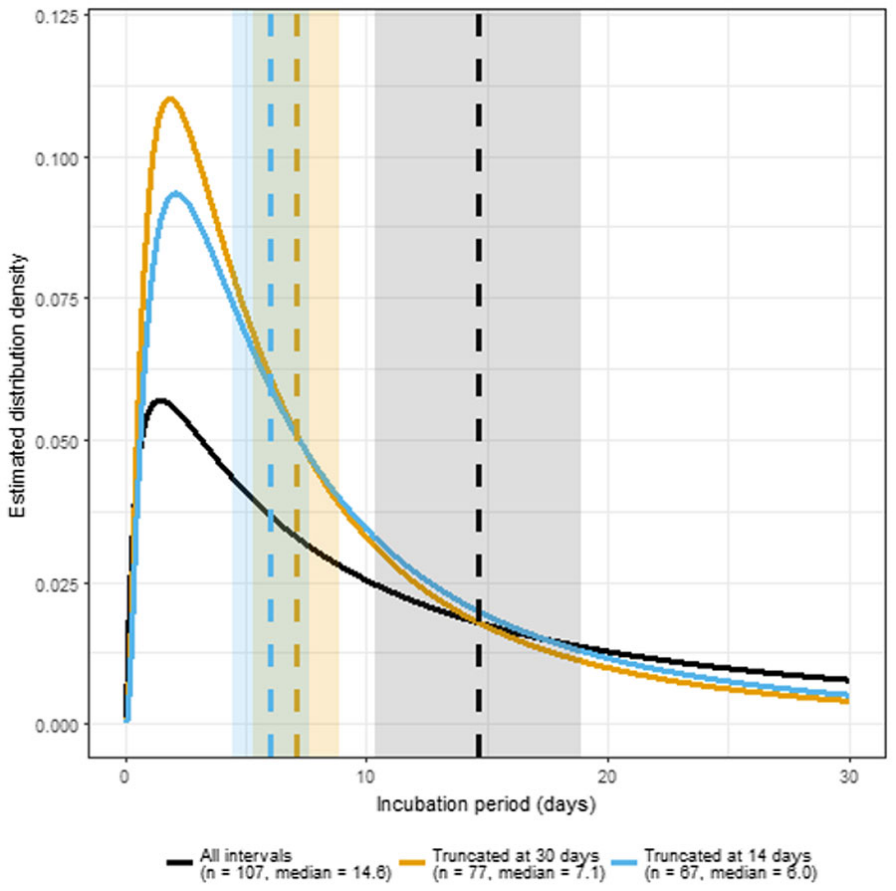
Estimated log-normal distributions of incubation periods for *Clostridioides difficile* infection (CDI) with median values and 95% confidence intervals indicated based on all patients with CDI after an initial negative culture and censored to exclude those with negative cultures collected more than 30 or 14 days prior to CDI diagnosis with no intervening culture collection.

Figure 3 shows the estimated log-normal distributions of incubation periods with median values and 95% confidence intervals indicated based on all patients with CDI after an initial negative culture and censored to exclude those with negative cultures collected more than 30 or more than 14 days prior to CDI diagnosis. Log-normal distributions provided a better model fit for the 3 populations than Gamma and Weibull distributions. When all patients were included the median incubation period was 14.6 days (range 1–165 days). When the data were censored to only include participants with negative cultures collected within 14 days, the estimated median incubation period was 6 days with 25% and 75% of estimated incubation periods occurring within 3 and 12 days, respectively. The estimated incubation periods did not differ significantly for patients classified as having probable (median, 14.8 days; 95% confidence interval [CI] 9.2–20.5), possible (median 11.6 days; 95% CI 4.9–18.3), or unlikely (median, 20.4; 95% CI 4.6–36.2) CDI (*P* > .05).

## Discussion

In a cohort of 4179 patients with negative cultures for toxigenic *C. difficile* on study enrollment, 107 (2.6%) were subsequently diagnosed as having CDI. The estimated median incubation period for CDI was 14.6 days when all patients were included but only 6 days when negative cultures collected more than 14 days prior to CDI diagnosis without intervening follow-up cultures were excluded. Despite weekly surveillance for rectal carriage of *C. difficile* while in healthcare facilities, asymptomatic colonization was detected prior to the diagnosis of CDI in only 17.8% of patients. These results suggest that the time from acquisition of colonization to the onset of symptoms was only a few days in many but not all CDI patients.

Our findings are consistent with the previous studies that suggested the incubation period for CDI is typically less than 1 week and is less than 2 weeks in most cases.^2,3^ The finding that the estimated incubation period is relatively short provides support for current surveillance definitions for CDI which implicate healthcare facilities as likely sites of acquisition of *C. difficile* in many cases diagnosed during admission or soon after discharge.^13^

Although the median incubation period was short, it is notable that there was a wide range in the calculated incubation period (ie, 1–165 days). The variability in the incubation period may reflect the fact that multiple hosts (eg, immune response to toxin, degree of alteration of the intestinal microbiota) and environmental factors (eg, antibiotic therapy, level of exposure to *C. difficile*) can impact the likelihood that a person acquiring colonization will develop a symptomatic infection.^1^ It is plausible that some patients acquiring toxigenic *C. difficile* maintain low levels of colonization until a factor such as antibiotic therapy promotes overgrowth with toxin production. For example, Figure 1. C shows the time line for a patient who maintained low levels of colonization with a ribotype F014-020 strain for several weeks but then developed CDI after receiving antibiotic therapy.

There is a concern that patients with asymptomatic carriage of toxigenic *C. difficile* on hospital or LTCF admission are at risk for a false-positive diagnosis of CDI if they develop diarrhea for other reasons and are tested for CDI.^5,9,16^ Similarly, it is plausible that some patients with new acquisition of colonization with toxigenic *C. difficile* could be falsely diagnosed as having CDI if they develop diarrhea due to other causes. In that regard, it is notable that 18.7% of patients diagnosed as having CDI in the current study were considered unlikely to have true CDI because they had >3 unformed stools per day but with a definite alternative explanation (eg, laxatives) or <3 unformed stools per day and no leukocytosis or radiographic findings consistent with CDI or ileus. Nevertheless, the observed estimated incubation periods did not differ significantly for patients classified as having probable, possible, or unlikely CDI.

Our study has some limitations. The study population was heterogeneous, and the results may not be generalizable to all populations. Although half of the CDI cases from the Cleveland sites were nursing home residents, we did not have information on nursing home residence prior to hospital admission for the Medical University of South Carolina. Thus, additional studies are needed to assess the incubation period for nursing home residents. Perirectal swabs were collected weekly only while participants were in healthcare facilities or when available after discharge during outpatient clinic visits or 2 of the study sites. Studies that include a more frequent collection of cultures are needed to provide a more accurate estimation of the incubation period. The day of CDI diagnosis was used for the calculation of the incubation period rather than the day of symptom onset. Thus, our results may overestimate the true incubation period if there are substantial delays between symptom onset and CDI diagnosis. Two of the 3 facilities used standalone NAAT tests for diagnosis which can lead to overdiagnosis of CDI due to detection of colonization.^9,11^ Finally, different screening methods were used at the different study facilities, and the percentage of patients receiving follow-up cultures was substantially lower at the Medical University of South Carolina than at the Cleveland sites.

In conclusion, our findings suggest that the incubation period for CDI is relatively short for most patients. These findings provide support for current surveillance definitions for CDI and have important implications for the control of *C. difficile.* Future studies are needed to identify interventions that reduce the risk of acquisition of *C. difficile* in healthcare settings.
